# Interplay between tunneling nanotubes and Wnt Signaling: Insights into cytoskeletal regulation and therapeutic potential

**DOI:** 10.1016/j.bbrep.2025.102065

**Published:** 2025-05-27

**Authors:** Tengfei Feng, Qi Xu, Shuangshuang Wang, Dongyu Hou, Xunwei Wu

**Affiliations:** a Savaid Stomatology School, Hangzhou Medical College, Hangzhou, Zhejiang, 311399, China; b Ningbo Stomatology Hospital, Ningbo, Zhejiang, 315000, China; c Engineering Laboratory for Biomaterials and Tissue Regeneration, Ningbo Stomatology Hospital, Ningbo, Zhejiang, 315000, China; d Ningbo Savaid Stomatology and Otolaryngology Medical Institute, Ningbo ENT Hospital, Ningbo, Zhejiang, 315000, China

**Keywords:** Tunneling nanotubes, Wnt pathway, Actin cytoskeleton

## Abstract

Tunneling nanotubes (TNTs) are membranous structures that enable direct intercellular transfer of mitochondria, proteins, RNAs, and signaling molecules, playing key roles in tissue repair, immune coordination, and stress adaptation. Among their critical functions, TNT-mediated mitochondrial transfer rescues metabolically impaired cells, yet the regulatory mechanisms governing TNT formation and function remain incompletely understood. Recent studies highlight the Wnt signaling pathway—a conserved regulator of cell fate, polarity, and cytoskeletal remodeling—as a central modulator of TNT dynamics. Through its canonical (Wnt/β-catenin) and non-canonical (Wnt/PCP and Wnt/Ca^2+^) branches, Wnt signaling orchestrates actin filament organization, bundling, and turnover, all of which are essential for TNT biogenesis and stability. This review critically examines the mechanistic intersection between Wnt signaling and TNTs, with an emphasis on how Wnt-driven cytoskeletal remodeling supports intercellular connectivity. Beyond basic mechanistic insights, we also explore the physiological and pathological relevance of this crosstalk—including its roles in tissue regeneration, immune modulation, cancer progression, and neurodegeneration. While the Wnt–TNT axis offers therapeutic promise, its context-dependent effects demand careful consideration.

## Highlights

• Wnt signaling orchestrates actin remodeling to regulate TNT formation and function.
• TNTs mediate mitochondrial transfer and signaling in regeneration, immunity, and cancer.
• Crosstalk between Wnt pathways and TNTs reveals novel therapeutic opportunities.

## Teaser

**One-Sentence Summary**: This review explores the interplay between Wnt signaling and tunneling nanotubes (TNTs), highlighting how Wnt-mediated cytoskeletal remodeling regulates TNT formation and function, with implications for tissue regeneration, immune modulation, and disease progression.

## 1 Introduction

Intercellular communication is a fundamental process that enables cells to coordinate responses, maintain tissue homeostasis, and adapt to environmental challenges. Among the various mechanisms of communication, tunneling nanotubes (TNTs) represent a unique form of long-distance, direct intercellular connectivity [1]. These dynamic, thin, membranous structures facilitate the transfer of diverse cellular cargo, including organelles [2], proteins [3], RNA [4], and signaling molecules [5], thereby playing critical roles in both physiological and pathological processes. Since their discovery in rat pheochromocytoma (PC12) cells [1], TNTs have been identified in a variety of cell types in vitro, including epithelial cells [6,7], immune cells [5,8,9], kidney cells [10], tumor cells [11,12], and neuronal cells [[13], [14], [15]] from different species origins. This suggests that TNTs are highly conserved in nature. In addition, TNTs have been recognized for their involvement in processes such as immune modulation [5], tissue regeneration [16], and cancer progression [17,18]. Understanding TNTs not only broadens our knowledge of intercellular communication but also opens doors to innovative therapeutic strategies targeting cellular networks.

A particularly significant function of TNTs is their ability to mediate mitochondrial transfer between cells [19]. This transfer can rescue metabolically compromised cells, promote survival, and enhance cellular adaptation under stress conditions [[20], [21], [22], [23]]. By directly exchanging mitochondria and other vital materials, TNTs help maintain tissue integrity and facilitate recovery in the face of damage or disease. Despite these important roles, the molecular mechanisms underlying TNTs formation, stabilization, and function remain incompletely understood. Focusing on these mechanisms is critical, as TNTs-mediated communication presents unique therapeutic opportunities in conditions such as cancer, neurodegeneration, and tissue repair.

Cytoskeletal dynamics, particularly the regulation of actin filaments, are central to TNTs biogenesis and function [1]. F-actin forms the core structural component of TNTs, supporting both their physical integrity and the transport of cargo [1]. Among the various pathways involved, the Wnt signaling pathway has emerged as a key modulator of cytoskeletal dynamics and cellular communication [24,25]. The Wnt pathway's canonical (Wnt/β-catenin) and non-canonical (Wnt/PCP and Wnt/Ca^2+^) branches regulate actin remodeling, essential for TNTs formation and stability. This review provides a novel perspective by integrating insights into TNTs biology with the regulatory influence of Wnt signaling, aiming to uncover their combined roles in tissue regeneration, immune modulation, and disease progression, and to highlight emerging opportunities for targeted therapeutic interventions.

## 2 Structural and functional aspects of TNTs

### 2.1 Structure and composition of TNTs

Tunneling nanotubes (TNTs) are highly specialized, dynamic structures that enable direct cell-to-cell communication [1,26]. Their morphology is defined by thin, membranous channels ranging from 50 to 700 nm in diameter, with lengths extending up to several cell diameters. These structures are delicate and transient, often breaking under physical stress, which highlights their dynamic and regulated nature [27]. TNTs can be broadly classified into two types: open-ended TNTs, which form continuous cytoplasmic bridges, and closed-ended TNTs, which establish contact between cells without direct cytoplasmic continuity [1,26].

**Fig. 1:**
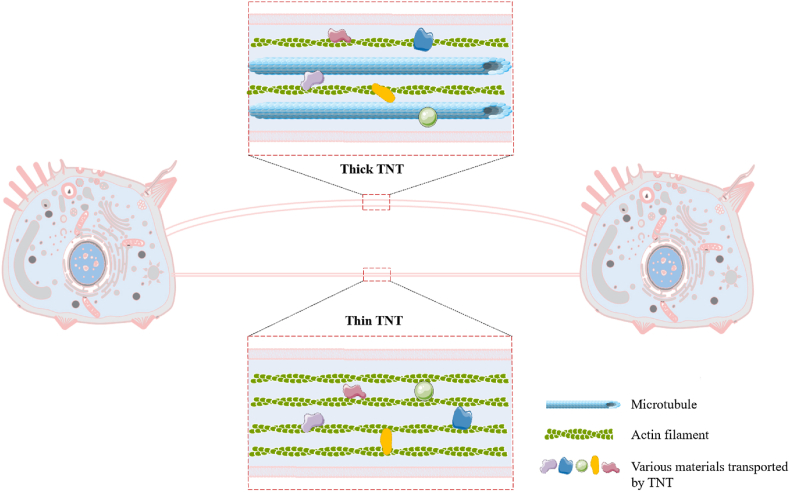
**A Schematic representation of thin and thick TNTs.** Thin TNTs contain only F-actin filaments, while thick TNTs have microtubules and actin backbones.

The cytoskeletal composition of TNTs is critical to their formation, stability, and function. The in vitro study demonstrated that the core component of TNTs is F-actin, which provides structural integrity and facilitates the transport of cellular cargo [1]. Thinner TNTs are primarily composed of actin filaments, while thicker TNTs may also incorporate microtubules or intermediate filaments [9] (Fig. 1). Microtubules are often associated with the transport of larger organelles such as mitochondria, lysosomes, and vesicles, while intermediate filaments provide additional mechanical support [9,28]. The precise composition of the cytoskeleton varies depending on the cell type, physiological conditions, and the specific cargo being transported.

TNTs formation occurs through two distinct mechanisms. The first involves the extension of filopodia-like protrusions from one cell toward another, which eventually fuse to form a TNTs [1]. The second mechanism involves the separation of two initially adjacent cells, leaving behind a tubular structure that bridges their cytoplasm [1]. Both processes depend on the dynamic remodeling of the actin cytoskeleton, highlighting the importance of actin regulatory proteins and signaling pathways in TNTs biogenesis.

### 2.2 Functions of TNTs

**Fig. 2:**
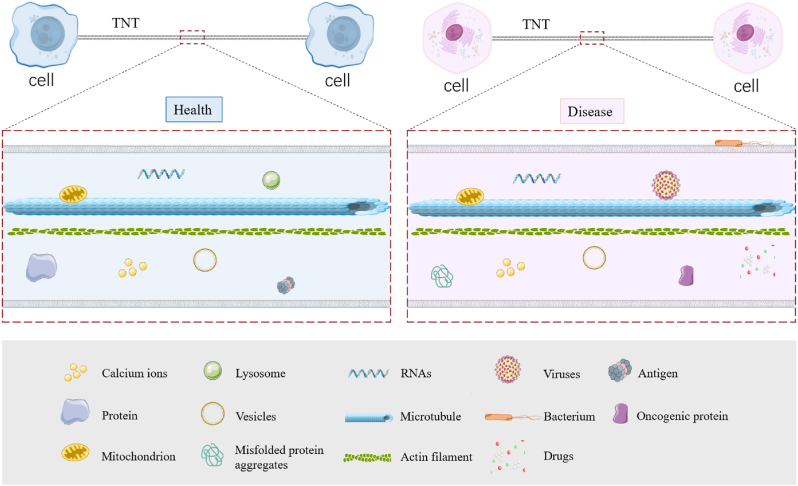
**A Schematic representation of different cargoes transported through TNTs between cells under either healthy (left) or diseased (right) conditions:** TNTs mediate the transfer of beneficial cargo such as mitochondria, proteins, calcium ions, and vesicles, supporting cellular homeostasis. In diseased states (right), TNTs facilitate the spread of harmful components like misfolded protein aggregates, viruses, bacteria, oncogenic proteins, and drug-resistant factors, contributing to disease progression.

Tunneling nanotubes (TNTs) play a pivotal role in intercellular communication by transferring organelles, proteins, RNAs, and signaling molecules across cells (Fig. 2). Emerging evidence suggests that the formation and function of TNTs are modulated by intracellular signaling pathways, notably Wnt signaling, which governs cytoskeletal remodeling essential for TNT dynamics.

#### 2.2.1 Mitochondrial transfer

Among the most studied TNT functions is the transfer of mitochondria between cells through the in vitro tissue culture study [2], a process that restores metabolic activity and promotes survival in stressed or damaged cells [[29], [30], [31]]. Recent findings suggest that Wnt signaling enhances TNT-mediated mitochondrial trafficking by regulating actin polymerization and motor proteins that drive mitochondrial motility [32]. For example, activation of Wnt/β-catenin signaling has been associated with upregulation of Connexin43 (Cx43), which facilitates both TNT formation and mitochondrial exchange in stem cell and immune contexts [33].

#### 2.2.2 Protein and RNA transfer

TNTs allow the transport of growth factors, transcription factors, microRNAs [34], and mRNAs [4], impacting cell fate decisions such as differentiation and proliferation. Wnt signaling, known for its role in gene regulation and cytoskeletal organization, may indirectly influence this function by modulating the stability and connectivity of TNTs, thereby affecting the efficiency of molecular exchange.

#### 2.2.3 Transfer of signaling molecules

The intercellular movement of calcium ions [5], inositol trisphosphate [35], and second messengers through TNTs allows coordinated signaling, especially in neurons and immune cells. Wnt/Ca^2+^ signaling is particularly relevant here, as it regulates intracellular calcium dynamics via effectors like CaMKII and PKC in vertebrate embryos [36], which also influence TNT formation and vesicle transport efficiency [37].

#### 2.2.4 Pathogen and drug resistance spread

TNTs can also propagate pathogenic materials such as viruses [38], bacteria [9], and drug-resistance proteins [39], contributing to disease progression. While this spread is largely facilitated by cytoskeletal remodeling, Wnt pathway dysregulation—common in cancer and infection—can further enhance TNT formation, thus accelerating the intercellular dissemination of deleterious factors [40,41].

In brief**,** in physiological conditions (Fig. 2, left), Wnt signaling supports TNT-mediated communication by promoting actin remodeling, enhancing the transport of beneficial cargo such as mitochondria, proteins, and vesicles. In pathological contexts (Fig. 2, right), aberrant Wnt activity may potentiate TNT-mediated transfer of oncogenic molecules, misfolded proteins, or pathogens, thereby contributing to disease progression. By serving as a molecular nexus, Wnt signaling shapes the structural and functional landscape of TNTs, highlighting the need to understand this interplay in both homeostasis and disease.

## 3 Regulation of TNTs formation by actin dynamics

### 3.1 Role of the actin cytoskeleton in TNTs

The actin cytoskeleton is fundamental to the formation, maintenance, and function of tunneling nanotubes (TNTs) [1]. F-actin, the filamentous form of actin, provides the structural backbone of TNTs and facilitates the directed transport of cargo along these structures [27]. The dynamic assembly and disassembly of actin filaments enable TNTs to extend, stabilize, and adapt to cellular needs and environmental cues.

Wnt signaling is a critical upstream regulator of these cytoskeletal dynamics, especially through its non-canonical branches (Wnt/PCP and Wnt/Ca^2+^), which modulate actin polymerization, bundling, and turnover. By activating small GTPases or calcium-sensitive proteins, Wnt pathways establish a permissive cytoskeletal environment for TNT initiation and elongation.

**Impact of Actin Polymerization Inhibitors:** Pharmacological agents such as latrunculin B [42] and cytochalasin D [43] that disrupt actin polymerization severely impair TNT formation and cargo trafficking [42], including mitochondrial transfer. These results underscore the dependency of TNTs on actin remodeling, a process in which Wnt signaling plays a facilitative and regulatory role.

### 3.2 Key regulators of actin dynamics and their Wnt-dependent modulation

#### 3.2.1 Arp2/3 complex and small GTPases (Cdc42, Rac1, RhoA)

The Arp2/3 complex initiates branched actin polymerization, which is essential for membrane protrusions such as filopodia and lamellipodia—precursors to TNTs [44]. Its activity is tightly controlled by small GTPases and their effectors [45,46], many of which are downstream targets or partners of Wnt signaling.

• **Cdc42**: Promotes filopodia-like TNT precursors [47]. It is activated downstream of the Wnt/PCP pathway and acts synergistically with Wnt effectors such as Dishevelled to enhance Arp2/3-mediated nucleation [46,48].
• **Rac1**: Facilitates lamellipodia formation and vesicle movement in TNTs. Wnt signaling enhances Rac1 activation through Dishevelled or cAMP/PKA pathways, thereby coordinating actin dynamics with intracellular signaling [47].
• **RhoA**: While typically suppressive of TNT formation via stress fiber induction, Wnt/Ca^2+^ signaling (via PKA) can inhibit RhoA-ROCK activity to favor TNT elongation [49,50].

#### 3.2.2 Actin-based motor: myosin X (Myo10)

Myo10 is essential for the extension and stabilization of TNTs [51]. Wnt/PCP signaling, through downstream activation of Cdc42 and cytoskeletal reorganization, may indirectly promote Myo10 recruitment to the plasma membrane and its interaction with actin filaments. This hierarchical coordination facilitates filopodial protrusion toward target cells [51].

#### 3.2.3 Scaffold protein: MSec and its effectors

MSec functions in membrane deformation and TNT assembly through its interaction with RalA and the exocyst complex [47]. While not directly a Wnt target, MSec-dependent TNT formation may be modulated by Wnt-induced cytoskeletal plasticity. For instance, Wnt/Ca^2+^ pathway-mediated CaMKII activation may enhance actin bundling and stabilize MSec-facilitated protrusions [52]. Additionally, Connexin43 (Cx43)—a Wnt/β-catenin target—can interface with both actin regulators and MSec to promote TNT stability and cargo transfer in stem and cancer cells, adding another layer of Wnt-mediated modulation [[53], [54], [55], [56], [57], [58], [59], [60]].

In summary, the intricate coordination of actin dynamics by Arp2/3, small GTPases, Myo10, and MSec forms the foundation for TNTs formation. Wnt signaling integrates into this framework by acting upstream or in parallel with these effectors—either by enhancing their expression (e.g., Cx43, Dvl1) or modulating their activity (e.g., Rac1, RhoA inhibition). These hierarchical and synergistic interactions position Wnt signaling as a key modulator of the cytoskeletal events that underlie TNT biogenesis and function.

## 4 Wnt signaling pathway in TNTs formation and function

### 4.1 Overview of Wnt signaling pathway

**Fig. 3:**
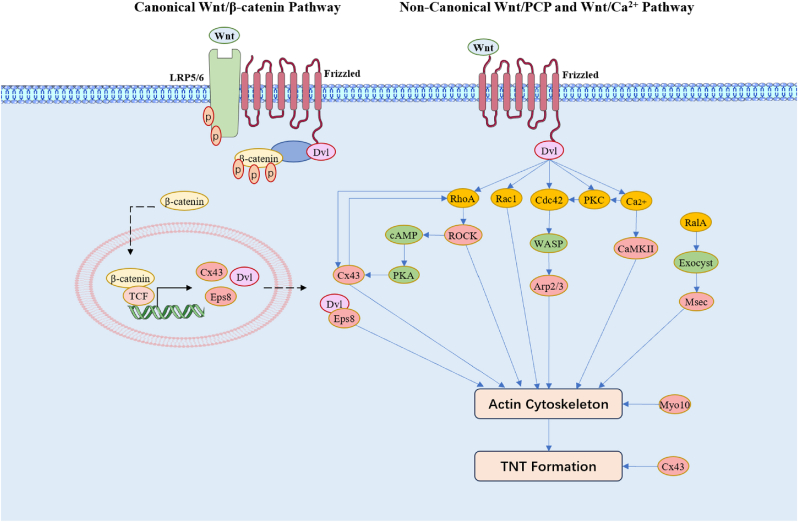
**Regulation of TNT Formation by Canonical and Non-Canonical Wnt Signaling Pathways:** This schematic illustrates how canonical (Wnt/β-catenin) and non-canonical (Wnt/PCP and Wnt/Ca^2+^) Wnt signaling regulate TNT formation via actin cytoskeleton remodeling. Canonical Wnt signaling promotes Cx43 and Eps8 expression, influencing actin dynamics. Non-canonical pathways regulate cytoskeletal organization through RhoA, Rac1, Cdc42, PKC, CaMKII, and the Exocyst complex. These signaling cascades coordinate actin polymerization, bundling, and structural integrity, ultimately promoting TNT formation and intercellular communication.

The Wnt signaling pathway regulates TNTs biogenesis through its canonical (Wnt/β-catenin) and non-canonical (Wnt/PCP and Wnt/Ca^2+^) branches. These pathways play distinct roles in modulating cytoskeletal remodeling [61], cellular signaling [62], and intercellular communication [63], all of which are pivotal for TNTs dynamics. The canonical Wnt/β-catenin pathway primarily focuses on transcriptional regulation of target genes that influence cytoskeletal organization, while the non-canonical pathways drive direct cytoskeletal rearrangements, polarity, and calcium signaling. Together, these branches integrate extracellular signals into cellular responses, facilitating the formation, stabilization, and functionality of TNTs (Fig. 3).

### 4.2 Wnt/β-catenin pathway

The canonical Wnt/β-catenin pathway is initiated by the binding of Wnt ligands to Frizzled receptors and LRP5/6 co-receptors, leading to the inhibition of the β-catenin destruction complex [64]. This stabilization of β-catenin allows its translocation into the nucleus, where it interacts with TCF/LEF transcription factors to regulate gene expression [65,66]. Wnt/β-catenin signaling has been shown to enhance actin dynamics during axon remodeling by upregulating key cytoskeletal regulators Dishevelled1 (Dvl1) and Eps8 in the culture of neuron cell isolated from newborn mice and E18 Rat embryos [67]. These proteins modulate actin filament nucleation and bundling, ensuring the structural integrity required for TNTs elongation and cargo transport [[68], [69], [70], [71]]. Additionally, β-catenin–mediated transcription promotes the expression of actin-associated proteins that stabilize TNTs structures under mechanical stress [69].

Additionally, studies in various cell types have demonstrated that the Wnt/β-catenin signaling pathway can upregulate *CX43* gene expression. This regulation occurs through the binding of β-catenin to TCF/β-catenin binding elements within the *CX43* promoter [53], indicating that CX43 is a typical target gene for Wnt signal transduction [[54], [55], [56], [57]].

Cx43 may have multiple roles in TNTs: Cx43 can directly regulate the formation and length of TNTs in breast cancer cells [58]; Cx43 facilitates TNT formation and mitochondrial transfer between human-induced pluripotent stem cell-derived mesenchymal stem cells and mouse epithelial cells [59]; Cx43 has also been shown to influence the number of TNTs in normal human trabecular mesh cells [60].

### 4.3 Wnt/PCP pathway

The planar cell polarity (PCP) branch of Wnt signaling is independent of β-catenin and involves the activation of small GTPases such as Rho, Rac, and Cdc42 [72]. These GTPases regulate the spatial organization of the actin cytoskeleton by balancing contractile and protrusive forces [73], both of which are crucial for TNTs formation. For example, **RhoA:** Promotes stress fiber formation and actomyosin contractility, which are important for stabilizing TNTs bases [74]; **Rac1**: Drives lamellipodia-like structures [73], enabling TNTs protrusion toward target cells. Rac1 can promotes actin polymerization [69], which is crucial for the formation of TNTs. Upregulating the Rac/F-actin pathway can lead to an increase in TNTs formation and subsequent increase in mitochondria transfer [75]; **Cdc42**: Facilitates filopodia formation, providing the initial scaffolds for TNTs development [76].

Additionally, the Wnt/PCP pathway can activate the cAMP/PKA (cyclic adenosine phosphate-dependent protein kinase) signaling pathway [77,78], which plays a crucial role in regulating the actin cytoskeleton and F-actin dynamics [79,80]. By phosphorylating and inhibiting RhoA membrane transposition and ROCK signaling, cAMP activation ultimately promotes the formation of TNTs [81].

Through these effectors, the Wnt/PCP pathway fine-tunes the dynamic remodeling of actin filaments, ensuring the efficient establishment and stabilization of TNTs.

### 4.4 Wnt/Ca^2+^ pathway

The Wnt/Ca^2+^ pathway modulates intracellular calcium levels [82], activating calcium-sensitive signaling molecules such as calmodulin-dependent protein kinase II (CaMKII) [83] and protein kinase C (PKC) [37]. These proteins regulate actin dynamics and TNTs stability through two primary mechanisms: (1)**Actin Bundling and Turnover**: CaMKII interacts with F-actin [[84], [85], [86]], promoting F-actin bundling and enhancing structural stability, and this activity may enhance TNTs resilience and ensure efficient cargo transport; (2) **Inhibition of Actin Polymerization**: By reducing the availability of G-actin monomers, CaMKII regulates the rate of actin turnover [87], preventing excessive elongation or instability of TNTs structures.

The most direct evidence that the Wnt/Ca^2+^ pathway regulates TNTs comes from the in vitro study by Vargas et al. [40]. Their study showed that activation of the Wnt/Ca^2+^ pathway upregulates TNTs formation in CAD cells and neurons by modulating the interaction between βCaMKII and actin, and increases TNTs-mediated interneuronal metastasis of vesicles as well as a-syn fibrils. Moreover, the actin-binding activity of βCaMKII is necessary to induce an increase in the number of TNTs between neuronal CAD cells, and this activity also helps to stabilize TNTs.

The dynamic interplay between the Wnt signaling pathway and TNTs demonstrates a synergistic regulation of cellular communication, cytoskeletal organization, and signal transduction. The three branches of the Wnt signaling pathway—Wnt/β-catenin, Wnt/PCP, and Wnt/Ca^2+^—contribute distinct but complementary mechanisms to TNTs dynamics (Fig. 3):

**Canonical Wnt/β-catenin Pathway:** By regulating actin polymerization through effectors like Dvl1 and Eps8, this pathway enhances actin filament assembly and stability [67], which are crucial for TNTs formation; β-catenin-mediated transcription further promotes the expression of genes (such as CX43 [53]) involved in cytoskeletal remodeling, enabling cells to adapt their structural frameworks to support TNTs-mediated intercellular communication.

**Non-Canonical Wnt/PCP Pathway:** The PCP pathway governs the spatial orientation and organization of actin filaments through small GTPases (Rho, Rac, and Cdc42) [72]. This regulation balances the contractile and protrusive forces needed for TNTs formation and stability, ensuring effective cargo transport.

**Non-Canonical Wnt/Ca^2+^ Pathway:** This pathway modulates intracellular calcium levels to activate CaMKII [83] and PKC [37,50], which play critical roles in actin filament bundling and turnover. These mechanisms stabilize TNTs while fine-tuning their dynamics to meet the demands of various cellular environments.

The integration of these pathways enables Wnt signaling to precisely regulate TNTs biogenesis, structure, and function, facilitating efficient intercellular communication.

## 5 Pathological and therapeutic implications of the TNT–Wnt interplay

The dynamic interaction between tunneling nanotubes (TNTs) and Wnt signaling plays a pivotal role not only in physiological processes such as tissue repair and immune coordination but also in pathological settings like cancer progression and neurodegeneration. Understanding this interplay provides valuable opportunities for targeted intervention.

### 5.1 Tissue regeneration

TNTs contribute to in vivo tissue repair by mediating the transfer of mitochondria and other reparative cargo from healthy to injured cells [88,89]. Wnt signaling—particularly the β-catenin pathway—supports stem cell activation and proliferation [90], and enhance TNTs-mediated a-syn fibrils and vesicle transfer [40]. This synergy could be exploited in regenerative medicine, for example by pharmacologically activating Wnt signaling to stimulate TNTs and accelerate recovery in injured tissues.

### 5.2 Immune modulation

TNTs facilitate the rapid exchange of signaling molecules, antigens, and mitochondria between immune cells [5,91,92], helping coordinate responses during infection or inflammation. Concurrently, Wnt signaling regulates immune cell activation, polarization, and migration [93], suggesting that Wnt activity may influence the formation and function of TNTs in immune settings. This dual regulation offers potential avenues to fine-tune immune responses or dampen excessive inflammation in autoimmune diseases.

### 5.3 Tumor progression

In vivo tumor models, TNTs allow tumor and stromal cells to exchange mitochondria, oncogenic proteins, and drug-resistance factors [39,[94], [95], [96]]. This intercellular network enhances metabolic plasticity and therapy evasion. Dysregulated Wnt signaling—frequent in many malignancies [52]—can potentiate TNT formation and function, reinforcing tumor adaptability. Therapeutic strategies targeting this axis may disrupt these communication networks, thereby sensitizing tumors to treatment.

### 5.4 Therapeutic targeting of the TNT–Wnt axis

• **Targeting Cytoskeletal Regulators:** Small-molecule inhibitors of actin remodeling proteins (e.g., Rho GTPases, Dishevelled) may selectively impair TNT formation in tumors or inflamed tissues [76].
• **Modulating Wnt Pathway Activity:** As Wnt dysregulation is implicated in cancer and fibrosis [97,98], pathway inhibitors (e.g., Porcupine or tankyrase inhibitors) may reduce TNT formation and intercellular signaling, restoring communication homeostasis.
• **Enhancing Regenerative Therapies:** Controlled activation of Wnt signaling in stem cells or therapeutic cell populations could increase TNT-mediated mitochondrial rescue, improving efficacy in wound healing, ischemic injury, or neuroregeneration.
• **Disrupting Pathogenic TNT Networks:** In conditions where TNTs propagate harmful agents (e.g., drug resistance, α-synuclein aggregates) [95,99], Wnt-targeting interventions may destabilize these conduits and enhance disease control.

**Fig. 4:**
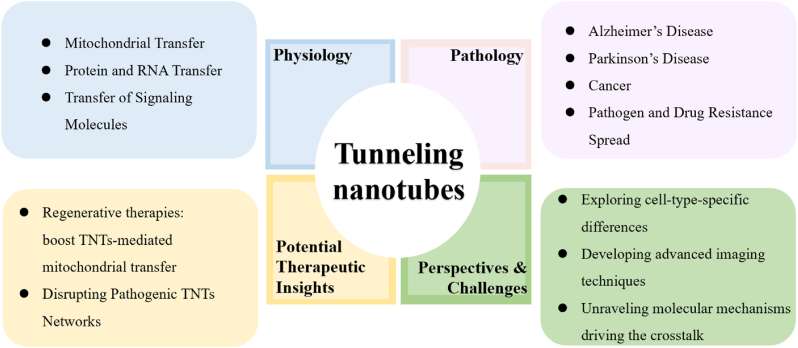
Graphical overview of TNT functions in the physiopathology, potential therapeutic insights, perspectives and challenges.

In summary**,** the TNT–Wnt axis constitutes a sophisticated communication network that bridges structural dynamics and intercellular signaling. By uncovering how these pathways cooperate across physiological and pathological contexts, we open new directions for therapy—whether by harnessing TNTs for tissue repair or inhibiting them to thwart tumor resistance. Future research should focus on refining Wnt-targeting strategies to modulate TNTs in a cell-type- and disease-specific manner, advancing the prospects of precision medicine (see Fig. 4).

In addition to the Wnt pathway, other signaling cascades also contribute to the regulation of TNT formation. For example, **Rho GTPases**, particularly **Cdc42** and **Rac1,** play pivotal roles in initiating and stabilizing TNT structures by modulating actin cytoskeletal dynamics [100]. Moreover, **MAPK signaling** has been implicated in promoting TNT formation in breast and ovarian cancer cells [101,102]. Notably, these signaling pathways may also interact with Wnt signaling in a coordinated manner to regulate TNT biogenesis, suggesting a complex, integrated regulatory network.

## 6 Risks and challenges of targeting Wnt signaling for therapeutic modulation of TNTs

While Wnt signaling presents an attractive therapeutic target due to its regulatory role in TNT formation, several challenges and risks must be considered to ensure translational relevance.

Wnt signaling plays divergent roles across tissues and disease states [103]. For example, while activation of the Wnt/β-catenin pathway may enhance tissue regeneration via TNT-mediated mitochondrial rescue, the same pathway can promote proliferation and immune evasion in cancers. Therefore, indiscriminate modulation may yield unintended consequences, including exacerbation of tumor growth or disruption of normal homeostasis.

Wnt pathways are essential for embryonic development, stem cell maintenance, and tissue architecture. Systemic inhibition or activation of Wnt signaling can interfere with these fundamental processes, potentially leading to adverse effects such as bone loss, gastrointestinal toxicity, or impaired organ regeneration. Long-term exposure to Wnt modulators has been associated with risks like fibrosis, organ dysfunction, or secondary neoplasia [104].

Wnt signaling intersects with immune regulatory circuits [105]. Pharmacologic manipulation may inadvertently activate pro-inflammatory responses or suppress necessary immune functions, increasing susceptibility to infection or autoimmunity. Additionally, current Wnt-targeting agents (e.g., Porcupine inhibitors, tankyrase inhibitors) may have off-target effects on unrelated signaling cascades due to limited pathway specificity [106,107].

Despite growing interest in Wnt-targeted therapies, their clinical translation faces significant hurdles. Many small-molecule inhibitors—such as Porcupine or tankyrase inhibitors—lack selectivity across the diverse family of Wnt ligands and Frizzled receptors, leading to potential off-target effects and toxicity [108]. Biologic agents like monoclonal antibodies (e.g., LRP6-targeting therapies) have shown promise but suffer from poor tissue penetration, particularly in dense or hypoxic environments like solid tumors or fibrotic wounds where TNTs are active [109]. Additionally, the temporal window of Wnt modulation is narrow; sustained inhibition or prolonged activation can reverse beneficial effects and promote adverse outcomes [110].

## 7 Conclusion and perspectives

This review highlights the pivotal role of tunneling nanotubes (TNTs) in intercellular communication and underscores the regulatory influence of Wnt signaling in TNTs biogenesis and function. By enabling the direct transfer of mitochondria, proteins, RNAs, and signaling molecules, TNTs contribute to tissue repair, immune coordination, and disease progression. Canonical Wnt/β-catenin signaling supports TNTs stability through transcriptional activation of cytoskeletal regulators, while non-canonical Wnt/PCP and Wnt/Ca^2+^ pathways fine-tune actin remodeling and intracellular dynamics to modulate TNTs formation and cargo transport.

The integration of TNTs dynamics with Wnt signaling unveils a critical regulatory axis with broad physiological and pathological implications. In regenerative medicine, activation of this axis may enhance cellular recovery and tissue repair. Conversely, in cancer or neurodegeneration, hyperactivation may exacerbate disease by facilitating deleterious cargo transfer. Thus, therapeutic interventions targeting the TNT–Wnt interface must balance efficacy with safety, given the Wnt pathway's essential role in development, immunity, and tissue homeostasis.

Future research should focus on three key areas: (1) elucidating cell-type-specific regulators of TNT–Wnt interplay, (2) developing in vivo imaging tools to visualize TNT–Wnt dynamics, and (3) defining molecular hierarchies that link Wnt effectors to TNT machinery. As illustrated in Fig. 4, integrating mechanistic insights with therapeutic perspectives will guide the design of targeted strategies to modulate TNT–Wnt signaling in a context-specific and temporally controlled manner, paving the way for innovative approaches in tissue regeneration, immunomodulation, and cancer therapy.

## CRediT authorship contribution statement

**Tengfei Feng:** Writing – original draft, Visualization, Formal analysis, Data curation. **Qi Xu:** Formal analysis, Data curation. **Shuangshuang Wang:** Visualization, Data curation. **Dongyu Hou:** Formal analysis, Data curation. **Xunwei Wu:** Writing – review & editing, Supervision, Funding acquisition.

## Funding

The work was supported by National Natural Science Foundations of China (82273554).

## Declaration of competing interest

On behalf of all authors, I declare that there are no conflicts of interest related to this manuscript. All authors have approved the manuscript and agree with its submission to *Biochemical and Biophysical Research Communications*.

## Data Availability

No data was used for the research described in the article.
